# Conjugative DNA Transfer Is Enhanced by Plasmid R1 Partitioning Proteins

**DOI:** 10.3389/fmolb.2016.00032

**Published:** 2016-07-19

**Authors:** Christian J. Gruber, Silvia Lang, Vinod K. H. Rajendra, Monika Nuk, Sandra Raffl, Joel F. Schildbach, Ellen L. Zechner

**Affiliations:** ^1^Institute of Molecular Biosciences, University of Graz, BioTechMed-GrazGraz, Austria; ^2^Department of Biology, Johns Hopkins UniversityBaltimore, MD, USA

**Keywords:** type IV secretion system, conjugative transfer, plasmid segregation, relaxase, pilus

## Abstract

Bacterial conjugation is a form of type IV secretion used to transport protein and DNA directly to recipient bacteria. The process is cell contact-dependent, yet the mechanisms enabling extracellular events to trigger plasmid transfer to begin inside the cell remain obscure. In this study of plasmid R1 we investigated the role of plasmid proteins in the initiation of gene transfer. We find that TraI, the central regulator of conjugative DNA processing, interacts physically, and functionally with the plasmid partitioning proteins ParM and ParR. These interactions stimulate TraI catalyzed relaxation of plasmid DNA *in vivo* and *in vitro* and increase ParM ATPase activity. ParM also binds the coupling protein TraD and VirB4-like channel ATPase TraC. Together, these protein-protein interactions probably act to co-localize the transfer components intracellularly and promote assembly of the conjugation machinery. Importantly these data also indicate that the continued association of ParM and ParR at the conjugative pore is necessary for plasmid transfer to start efficiently. Moreover, the conjugative pilus and underlying secretion machinery assembled in the absence of Par proteins mediate poor biofilm formation and are completely dysfunctional for pilus specific R17 bacteriophage uptake. Thus, functional integration of Par components at the interface of relaxosome, coupling protein, and channel ATPases appears important for an optimal conformation and effective activation of the transfer machinery. We conclude that low copy plasmid R1 has evolved an active segregation system that optimizes both its vertical and lateral modes of dissemination.

## Introduction

Extrachromosomal DNA elements such as plasmids are responsible for their own propagation in dividing host cells. Low copy number plasmids rely on active segregation mechanisms for stable inheritance. In addition, many acquire the capacity for horizontal dissemination via bacterial conjugation. Because bacterial resistance to antibiotics is an immense problem in human health, research has focused on gaining detailed knowledge of the initiation stage of conjugation and its control. The process has been best studied in Gram-negative organisms where multiple mating pore formation (Mpf) proteins assemble a cell envelope spanning transport channel and cell surface pili or adhesins mediate contact between cells. A receptor, called the type IV coupling protein (T4CP), is positioned at the cytoplasmic entrance of the secretion channel to recognize specific plasmid-bound protein complexes and deliver them to the channel. Following an initiation signal that has never been defined, the nucleoprotein cargo is then pumped through the transport apparatus in a reaction requiring ATP.

Regulation of conjugation involves donor cell perception of environmental signals. Knowledge of the control circuits coupling extracellular quorum signals and other stimuli to transcription of conjugation genes is increasing (Frost and Koraimann, 2010; Christie and Gordon, 2014; Clewell et al., 2014; Gibert et al., 2014). Yet, it remains challenging to discover how a potential recipient cell stimulates donor conjugative DNA transfer upon cell contact. We have postulated that bacteriophage might mimic potential recipient cells and initiate a signaling pathway that activates mechanisms typically involved in gene transfer. Thus, studies of bacteriophage that exploit conjugative pili as receptors for penetration of host cells are a promising approach to discover how cell contact-activated regulation of a type IV apparatus might operate.

Our work with the group 1 RNA phage R17 and the IncFII paradigm conjugation system R1 (Lang et al., 2011; Lang and Zechner, 2012) established that infection of the host required not only pilus biogenesis factors including TraA pilin, the Mpf proteins, the lytic transglycosylase P19 and the T4CP ATPase, but additionally the relaxosome, a complex of proteins generally required for binding and preparing the plasmid DNA origin of transfer (*oriT*) for export to recipient cells. The relaxosomes of F-like systems are well characterized (de la Cruz et al., 2010; Zechner et al., 2012). TraI is a bifunctional relaxase that cleaves one plasmid strand at *oriT* forming a covalent linkage to the nicked strand in the process (Matson et al., 1993). Recognition motifs enable TraI to bind the T4CP receptor and secretion of the TraI-DNA adduct delivers the plasmid to the recipient (Lang et al., 2010). A distinct functional region of TraI provides the essential helicase activity to generate single-stranded DNA (ssDNA) for export (Matson et al., 2001). In contrast to conjugative DNA transfer, R17 uptake via the R1-16 type IV apparatus does not require the entire TraI protein. This finding allowed us to define a novel domain of TraI necessary for activation of the nucleoprotein transfer via phage-generated signals (Lang et al., 2011). This work and previous biochemical studies support a model where the T4CP has a key role in coupling perceived signals of extracellular origin with intracellular cues provided by the relaxosome to activate the type IV channel (Berry and Christie, 2011; Lang et al., 2011). A following study showed that the activation domain of TraI is not only crucial to priming the T4CP for phage and conjugative transfer but also in signaling activation of the transporter for mobilization of competing plasmids such as ColE1 under conditions where the conjugative R1-16 plasmid is transfer deficient (Lang et al., 2014).

Another general function of conjugative pili is to form contacts with other cells and abiotic surfaces to promote biofilm development (Ghigo, 2001). Studies investigating the underlying mechanisms using F-like plasmids have highlighted the importance of pilus structure (Ghigo, 2001; Reisner et al., 2003). The *E. coli* biofilm phenotype and pilus-specific phage sensitivity can therefore be combined with general mutagenesis to identify proteins of host or plasmid origin that alter the conformation or function of the envelope spanning apparatus. Using a screen of this type we identified a miniTn5 mutant derivative of plasmid R1-16, which assembled conjugation machinery able to transfer DNA with wild type efficiency yet the pili promoted poor biofilm formation and were completely deficient for R17 phage infection even with overnight incubation (Nuk et al., 2011). Surprisingly, the site of transposon insertion was the R1-16 parMRC operon, which is involved in active segregation (partitioning) of the low copy plasmid. The system involves a centromere-like sequence *parC* bound by the adapter protein ParR and the actin-like ATPase ParM to form bipolar spindles, which push sister plasmids to the cell poles during cell division (Moller-Jensen et al., 2003; Salje and Lowe, 2008; Bharat et al., 2015). Segregation systems like parMRC are key to faithful plasmid inheritance. Moreover, type I ParA-like proteins of plasmid and chromosomal origin are also involved in intracellular partitioning of cellular organelles and proteins (Lutkenhaus, 2012; Roberts et al., 2012; Jones and Armitage, 2015). A connection between plasmid partitioning factors and DNA transfer machinery was established for the tumor-inducing (Ti) plasmid of *Agrobacterium tumefaciens*. In that system the ParA-like protein VirC1 spatially coordinates early DNA transfer events by mediating interactions between the T4CP VirD4 and the relaxase VirD2-DNA transfer intermediate (Atmakuri et al., 2007).

In this study we investigate the contribution of the R1 partitioning proteins ParM and ParR to the nucleoprotein transfer activities of the plasmid type IV secretion system (T4SS).

## Results

### *parMRC* mutation blocks R17 adherence and delays transfer initiation

**Figure 1:**
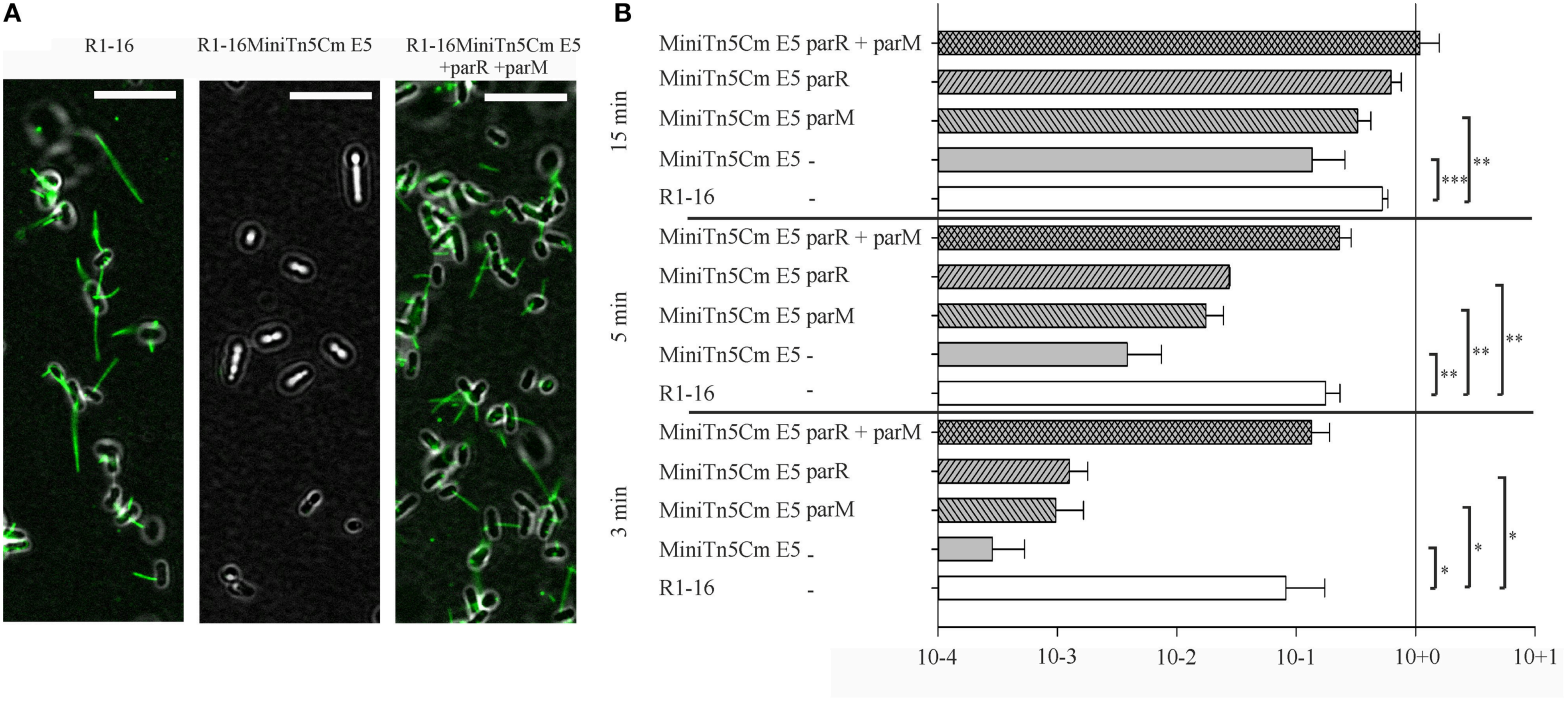
***parMRC* mutation blocks R17 adherence and delays transfer initiation. (A)** *E. coli* MS411 carrying R1-16 or R1-16MiniTn5 with and without complementation plasmids were incubated with Alexa488-labeled R17 phage. Phage decorated pili (green) are visualized by fluorescence microscopy. Phage binding to R1-16 (left panel) ceases in the MiniTn5 E5 mutant (middle panel) and is restored when *parM* and *parR* are provided *in trans* (right panel). Scale bar = 10 μm. **(B)** *E. coli* MS411 [R1–16] and MS411 [R1-16miniTn5 Cm E5] were mixed with plasmid free *E. coli* MS614. Conjugation was stopped after 3, 5, and 15 min and the number of transconjugant cells per donor that had acquired the plasmid was determined by selective plating. Vectors expressing *par* genes in trans (as labeled, y-axis) were used for complementation. The lower than wild type efficiency of R1-16miniTn5 transfer was rescued at all time points by the combination of *parM* and *parR in trans.* Standard deviations are shown, n = 3, significance was determined using a one-sided *t*-test, *P < 0.05; **P < 0.005; ***P < 0.001.

Mutagenesis of plasmid R1-16 used the transposon delivery system pUT-miniTn5Cm (Nuk et al., 2011). A selection step requiring conjugative transfer of the R1-16 mutant derivatives was included to eliminate those with transposon insertions in the plasmid *tra* genes. One biofilm deficient mutant, R1-16miniTn5Cm E5, carried the transposon inserted at position 488 of *parM* (Accession Number X04268), effectively blocking transcription of *parM* and *parR.* Disruption of this locus did not lead to an immediate loss of plasmid from the population and donor cultures conjugated normally in a standard 30 min mating experiment (Nuk et al., 2011) Nonetheless, the poor biofilm formation and the complete R17 resistance of hosts carrying this mutant suggested that the parMRC locus could be involved in the assembly and function of the conjugation machinery. We first asked whether conjugative pili assembled in the absence of *parMRC* were defective for bacteriophage attachment. *E. coli* MS411 carrying R1-16 or R1-16miniTn5Cm E5 were combined with fluorescently labeled R17 and visualized microscopically (Figure 1A). Attachment of the labeled R17 to wild type pili was apparent but strikingly absent from hosts carrying the mutant. The attachment defect was complemented by expression of *parM* and *parR in trans*. These data suggest that pili assembled in the absence of *parMRC* have an abnormal conformation.

We then asked whether a defect in early stages of plasmid transfer could be detected. *E. coli* MS411 [R1-16miniTn5Cm E5] donors were combined with *E. coli* MS614 recipient cells in broth culture. Conjugation was interrupted after 3–15 min and transconjugants were selected on agar plates. After 3 min of coincubation, transfer of the R1-16miniTn5Cm E5 was detected, but at frequencies 2–3 log units lower than transfer of wild type R1-16 (Figure 1B). Complete complementation of transconjugant formation at this time point was observed by providing *parM* and *parR* in combination on an expression plasmid *in trans*. Presence of either *parM* or *parR* was not sufficient. Significantly lower transfer frequencies were also observed for R1-16miniTn5Cm E5 compared to wild type after 5 and 15 min of conjugation. The magnitude of this difference decreased with increasing time, however. In every case, addition of *parM* and *parR in trans* fully complemented mating efficiency to wild type levels. We conclude that *E. coli* carrying the *parMRC* transposon insertion in R1-16 exhibits a delayed initiation of transfer phenotype that is fully overcome when cultures are allowed to conjugate for longer than 15 min.

### *parMRC* disruption reduced *oriT* cleavage *In vivo*

**Figure 2:**
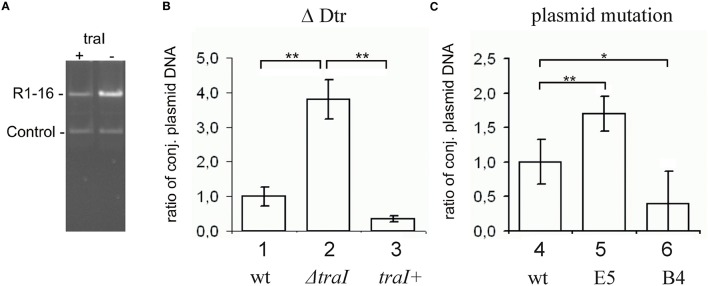
**Change in plasmid copy numbers or *oriT* cleavage activity *in vivo* altered yields of R1-16 DNA. (A)** R1-16 or mutant derivatives were co-maintained in *E. coli* cells with a second control replicon. The yield of the R1-16 and the copurified control DNA was determined following *Xba*I cleavage and gel resolution by measuring band intensity with Image J. **(B,C)** The yield of the R1-16 derivative was normalized by the yield of control plasmid. For each condition compared [loss of DNA processing, ΔDtr **(B)**, or transposon insertion in a stability locus **(C)**], the normalized value of R1-16 DNA (set to 1) in lanes 1, 4 was compared to the yield after the indicated variation. Plasmid yield varied with loss (lane 2) or gain (lane 3) of *traI* expression in trans to a R1-16*traI* mutant derivative; or due to the transposon carrying mutants indicated (lanes 5,6). Significant differences are shown, *n* = 3, significance was determined using a one-sided *t*-test, *p < 0.05; **p < 0.01.

Transfer initiation requires the activity of the DNA processing relaxosome complex. We asked next whether the *parMRC* disruption influences nicking of R1-16 *oriT* by TraI *in vivo*. R1-16 plasmids express conjugative genes constitutively and therefore support continuous relaxosome assembly. Within the relaxosome TraI maintains an equilibrium of cleaving and resealing of the nick site, meaning that a fraction of R1-16 will be in a nicked state at any moment (Zechner et al., 1997). When host cells are lysed for plasmid DNA isolation, the population of nicked molecules covalently attached to TraI should be lost during phenol extraction, lowering the yield. In contrast, any condition that disrupts *oriT* cleavage would allow R1-16 to remain supercoiled, increasing DNA recovery. To validate this assay of relative plasmid yield we combined R1-16 or mutant derivatives in *E. coli* M1174 cells with a second independent replicon. The two plasmids were copurified by phenol extraction, linearized once with *XbaI* and applied to agarose gels to detect quantitative variation in the apparent copy number of the conjugative plasmid relative to the second replicon. Figure 2 illustrates changes in plasmid ratios obtained by controlled manipulation of *oriT* DNA processing (Δ Dtr) through deletion of *traI*. The band intensities of the recovered R1-16 derivatives were normalized to the internal control plasmid and compared (Figures 2A,B). Values obtained for R1-16 wild type plasmid were set to 1. Disruption of the *traI* gene resulted in a nearly four-fold higher relative yield compared to wild type R1-16 DNA. Induction of *traI* expression *in trans* restored nicking activity, resulting in a plasmid yield significantly lower than in the absence of *traI*. We have used a similar assay previously (Nuk et al., 2011) to test whether random insertions of transposon miniTn5 in plasmid R1-16 result in plasmid instability. Validation of the assay in that case relied on controlled manipulation of the plasmid R1 copy number control system. In the current study, the plasmid recovery assay was applied to *E. coli* cells carrying R1-16 wild type, R1-16miniTn5Cm E5 and, as positive control, an additional transposon insertion in the *yjcA* gene close to the *kis*/*kid* stability locus of plasmid R1. Due to the insertion in the *parMRC* locus, we predicted relatively low yields of R1-16miniTn5Cm E5 compared to the reference replicon (Jensen and Gerdes, 1999), yet surprisingly the *parMRC* mutant derivative was obtained in higher relative yields than wild type R1-16 (Figure 2C). In comparison the control mutant B4, carrying the miniTn5 at the *kis*/*kid* locus, was poorly recovered, as expected for a destabilized plasmid. A possible explanation for the unexpectedly high yield of the *parMRC* mutant is that the assay outcome reflects a stronger defect in relaxosome activity than in plasmid stability. In that case, the partitioning components of the ParMR*C* system appear to enhance *oriT* DNA processing *in vivo*.

### The relaxase of TraI is stimulated by ParM and ParR *In vitro*

**Figure 3:**
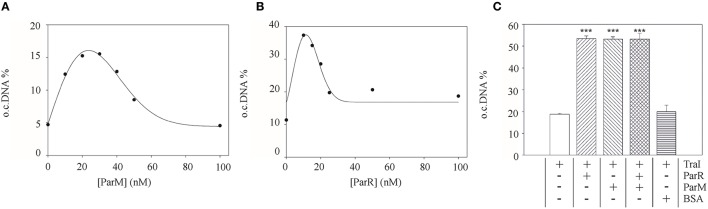
**Purified ParM and ParR stimulate TraI relaxase activity**. An *oriT*-carrying plasmid substrate was preincubated with increasing concentrations of ParM or ParR before addition of 25 nM TraI. The yield of open circular DNA relative to supercoiled substrate after 20 min reaction was visualized by agarose gel electrophoresis and band intensity measured with ImageJ. Stimulation of TraI relaxase activity by ParM alone **(A)** or ParR alone **(B)** is shown with representative curves. **(C)** Stimulation of TraI (25 nM) was measured with ParM (15 nM) or ParR (15 nM) alone and in combination. BSA (500 nM) served as negative control. Standard deviations are shown, *n* = 3, significance was determined using a one-sided *t*-test, ***P < 0.001.

To test whether this effect of the partitioning components directly involves TraI we purified ParR and ParM proteins and measured the impact of these effectors on known enzymatic activities of the TraI enzyme *in vitro*. A standard *oriT* DNA-cleavage assay used to monitor relaxase enzyme activity measures the conversion of supercoiled plasmid substrate to the open circular form using agarose gel electrophoresis (Lanka and Wilkins, 1995; Csitkovits et al., 2004). A supercoiled substrate plasmid (4 nM) carrying 420 bp of R1 *oriT* (pDE100) was preincubated with putative effector proteins ParM or ParR and the reaction started by the addition of 25 nM TraI (Figure 3). The percentage of *oriT* DNA captured in open circular form was significantly enhanced by the additional presence of ParM or ParR in a concentration dependent manner. Maximum stimulation (~three-fold, 5–16%) was observed when ParM was present in equimolar amounts (20–30 nM) relative to TraI. ParR alone (10 nM) stimulated TraI relaxase activity nearly four-fold (11–38%). At higher concentrations ParM and ParR failed to stimulate. Moreover, no superstimulation was observed when both factors were present. We then asked whether the Par proteins also stimulate truncated versions of TraI in this assay (not shown). N-terminal fragment TraI _1–308_ (TraIN308) forms the minimal relaxase domain, and residues 1–992 (TraIN992) comprise the relaxase and the complete activation domain absolutely required for all T4SS activities we have tested thus far. Titration of either Par effector protein to the reactions containing truncated forms of TraI did not result in stimulation of *oriT* cleavage. We conclude therefore, that both ParM and ParR stimulate the *oriT* cleaving and joining activity of TraI independently. Stimulation was observed exclusively with the full length TraI protein.

#### ParM and ParR mediated stimulation of TraI is specific for the relaxase reaction

In addition to DNA transesterase activity TraI also acts as a single-stranded (ss)DNA dependent ATPase and helicase that unwinds the plasmid DNA duplex in preparation for transfer to recipient cells. We next asked whether the Par proteins affect these enzyme activities of TraI. We measured ATP hydrolysis by purified TraI on single-stranded circular M13 DNA in the absence or presence of increasing concentrations of ParM and ParR. The specific activity of TraI was 226 kmol ATP/h/mol protein. No stimulation of this activity was observed with additional proteins present.

We next tested whether the Par proteins affect the helicase activity of TraI. The enzyme initiates unwinding on any DNA substrate if it is able to first bind to a stretch of ssDNA 5′ to the duplex junction (Kuhn et al., 1979; Csitkovits and Zechner, 2003). We generated two dsDNA substrates with a 60 bp central region of unwound DNA to support helicase loading (Sut et al., 2009). The substrates contained R1 *oriT* DNA for specific TraI binding (Williams and Schildbach, 2006) or non-specific sequences. The extent of DNA unwinding on these substrates agreed well with our previous results (Sut et al., 2009). However, unlike the stimulatory effect we obtain with the auxiliary effectors TraM, IHF, or TraD, no enhancement of helicase activity was observed in the additional presence of ParM or ParR alone, or in combination, under any conditions we tested (not shown). These findings indicate that the effects of Par proteins on TraI are specific for the enzyme's relaxase activity.

#### TraI stimulates ParM ATPase

**Figure 4:**
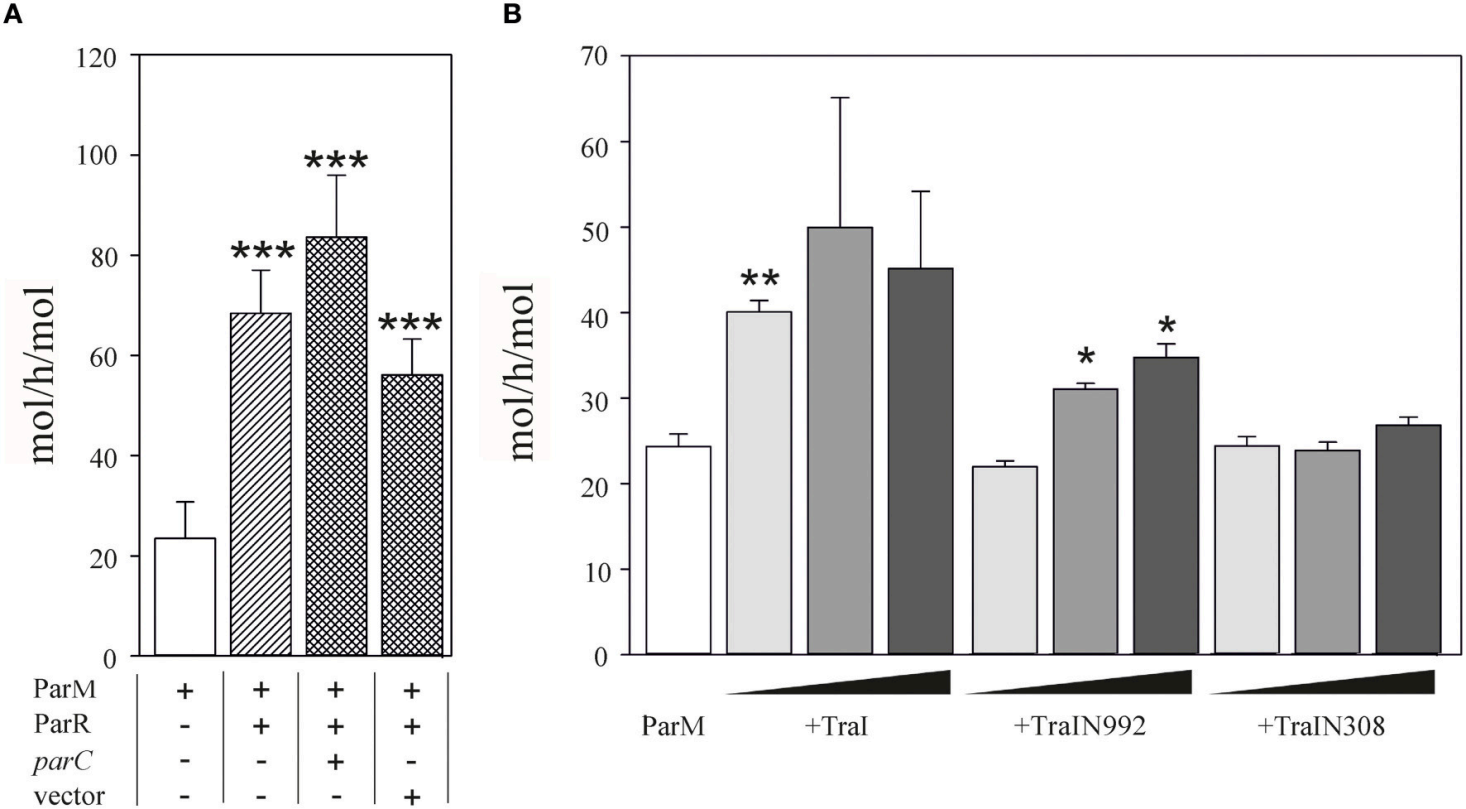
**ATPase activity of ParM is stimulated by TraI. (A)** The ATPase activity (mol h^−1^ mol^−1^) of 500 nM ParM was determined in a colorimetric assay measuring free orthophosphate released by ParM in the presence or absence of ParR and the presence of plasmid carrying *parC* DNA or a control DNA lacking *parC* (pDE110). **(B)** Effect of increasing (10, 50, 100 nM) concentrations of effector protein TraI, TraIN992 or TraIN308 on ATP hydrolysis by ParM (500 nM) is shown (mol h^−1^ mol^−1^). Standard deviations are indicated, *n* = 3, significance was determined using a one-sided *t*-test, *P < 0.03; **P < 0.003; ***P < 0.001.

We then performed the reciprocal test and asked whether TraI could stimulate ParM ATPase (Figure 4). Using conditions standardized by the K. Gerdes laboratory we measured 23.5 ± 7.3 mol ATP hydrolysed per hour per mol ParM (mol/h/mol). In good agreement with Jensen et al. (Jensen and Gerdes, 1997), this activity was increased three-fold in the additional presence of excess ParR (9 mM). ParR mediated stimulation was increased to ~four-fold in the additional presence of 17 nM double-stranded (ds)DNA containing *parC*. No additional ParR enhancement of ParM ATPase was observed when the dsDNA lacked *parC*. The effect of TraI protein on ParM ATPase was then tested using increasing concentrations of the full length TraI, N-terminal fragments TraIN992 and TraIN308 (Figure 4B). Significant stimulation of ParM ATPase was observed with the full length TraI and the TraIN992 fragment but not by the smallest TraIN308 variant. No ssDNA effector was present in the reaction mixtures effectively silencing ATP hydrolysis by TraI itself. Moreover, TraIN992 lacks ATPase activity (Matson and Ragonese, 2005). We conclude that TraI increased ATP hydrolysis by ParM.

Taken together these results imply that the mutual stimulation of ParM ATPase and TraI DNA transesterase activities is due to protein-protein interactions supported most efficiently with the full length TraI protein.

#### Par protein-TraI interactions do not alter DNA binding activities

**Figure 5:**
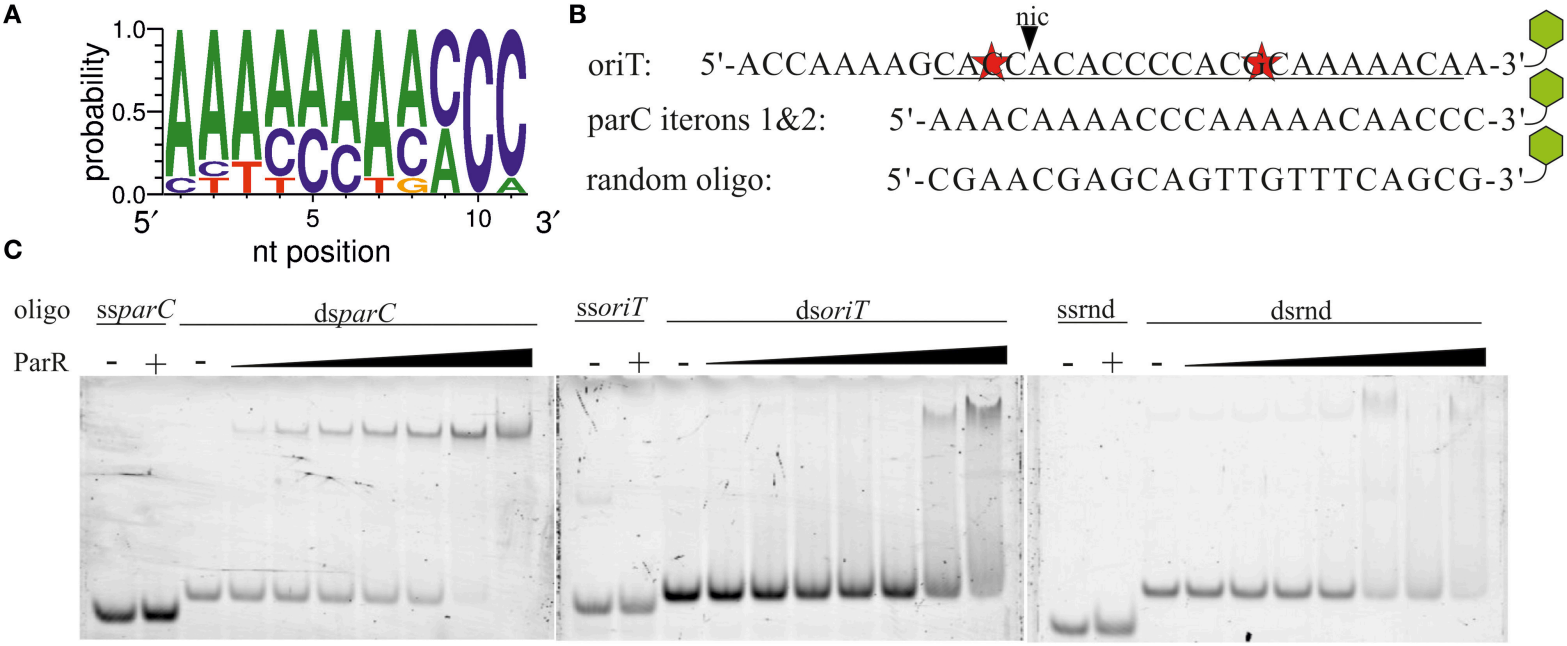
**ParR does not specifically bind to *oriT.* (A)** Consensus sequence of the *parC* iterons. **(B)** The sequence of R1 *oriT* at *nic* (black triangle) resembles the *parC* site. Underlined bases represent the best match of two *parC-*like iterons that contain two mismatches (red stars). Remaining bases are part of less ideal pseudo-iteron sequences. *parC* iterons 1 and 2. A random oligo sequence was created as control substrate. Green hexagons symbolize a 3′-TAMRA label. **(C)** Increasing concentrations of ParR (20–320 nM) were combined with 4 nM of TAMRA-labeled single- and double-stranded variants of *oriT* (ss-, ds*oriT*), *parC* iterons 1&2 (ss-, ds*parC*), and a random oligo (ss-, dsrnd). Protein—DNA complexes were resolved from free DNA by EMSA.

All of the proteins known to stimulate either the relaxase or helicase activities of TraI (Mihajlovic et al., 2009; Sut et al., 2009) bind to *oriT* DNA, specifically, in the case of TraM, TraY, and IHF or, in the case of the coupling protein TraD, in a sequence independent manner (Tsai et al., 1990; Nelson et al., 1995; Verdino et al., 1999; Schröder et al., 2002; Wong et al., 2011). ParR binds specifically to two sets of five direct repeats at the *parC* site (Moller-Jensen et al., 2003, 2007). These authors also showed that a minimum of two iterons is sufficient to support ParR binding (Moller-Jensen et al., 2003). We noted that *oriT* of plasmid R1 contains an A/C rich sequence that may constitute two *parC*-like iterons with a single mismatch to the consensus in each (Figure 5). The *parC*-like sequence overlaps part of the inverted repeat and neighboring bases specifically recognized by TraI (Stern and Schildbach, 2001) raising the possibility that ParR stimulates TraI activity through *oriT* binding. We compared ParR binding to different DNA fragments using an electrophoretic mobility shift assay (EMSA) (Figure 5). No binding of ParR to any ssDNA was observed. As a positive dsDNA control, a 22 bp fragment containing iterons 1 and 2 of *parC* was used. Mobility shift of the *parC* fragment was observed beginning at protein/DNA molar ratios of five to one, comparable to published values (Moller-Jensen et al., 2003). By contrast a mobility shift of the *oriT* sequence by ParR required a 40-fold molar excess of protein to DNA, equivalent to amounts necessary to shift a random-sequence substrate that served as negative control. We conclude that ParR does not specifically bind *oriT*.

Due to this finding, we next asked whether the Par proteins alter TraI-DNA interactions. We tested two hypotheses: Par-mediated stimulation of relaxase activity is due to (i) a higher rate of TraI association with the substrate or (ii) a stabilization of the product in a cleaved state. Fluorescence intensity and anisotropy measurements of TraI association with a 3′-TAMRA labeled 17mer *nic*-substrate have been described in detail (Stern and Schildbach, 2001; Harley et al., 2002; Williams and Schildbach, 2006; Hekman et al., 2008; Dostal and Schildbach, 2010). In a set of experiments using this approach (Figure S1) we investigated whether ParR or ParM induce variation in TraI DNA binding. K_D_s for TraI, TraIN308 (relaxase domain), and TraIΔN308 (helicase domain) were determined. The presence of ParM or ParR (both 10 nM) did not change affinity of TraI for *oriT*-DNA. Thus, we conclude that the partitioning factors do not stimulate the relaxase reaction by altering the enzyme's DNA binding properties (see Supplementary Material for full description of results).

### ParM binds conjugation proteins *In vivo*

**Figure 6:**
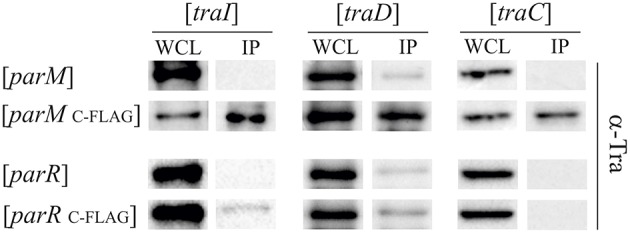
**ParM binds ATPases TraI, TraD, and TraC *in vivo***. *E. coli* MS411 cells carrying plasmids with the indicated *tra*-genes (above) and coexpressing FLAG-tagged or wild type *par*- alleles (left) were lysed and treated with formaldehyde to cross-link interacting proteins. Co-retention of Par protein-binding partners on FLAG-affinity matrix was monitored by Western analysis. Antibodies to the transfer proteins indicated were used for detection in whole cell lysates (WCL) and in the fractions retained on FLAG affinity beads (IP).

Given that ParM and ParR increase TraI relaxase activity but do not bind to *oriT* DNA we next tested for direct protein-protein interactions. Par protein fusions were engineered with terminal FLAG-epitopes. Expression plasmids for the tagged fusion proteins were maintained in *E. coli* MS411 cells carrying either R1-16, or a second vector expressing a candidate *tra* gene. Following induction of fusion protein expression cells were lysed, protein complexes briefly cross-linked with formaldehyde and the Par proteins captured on FLAG-affinity beads. Bound proteins and their specific interaction partners were washed, eluted, and visualized by western immunoblotting. Based on the phenotypic and biochemical results, candidates for specific Par protein binding included TraI and the second key factor involved in pilus specific R17 phage infection, T4CP TraD, as well as the VirB4-like ATPase TraC, which is essential for assembly and function of R1 pili. Tra proteins retained by Par proteins on the FLAG affinity matrix were detected with polyclonal antibodies generated to specific transfer proteins. The amounts of Tra proteins detected in the whole cell lysates reflect native levels produced from R1-16. The Tra specific antibodies revealed that TraI, TraD and TraC were co-retained by affinity beads together with ParM_C−FLAG_ (Figure S2). The specificity of these interactions was confirmed with a *par* allele lacking FLAG. In contrast ParR retained very small amounts of TraI and TraC but only with the C- terminal FLAG epitope (Figure S2). To assess whether the observed Tra-Par binding interactions can occur in the absence of the other segregation and transfer components, we co-produced the proteins in a pairwise manner. As shown in Figure 6, TraI, TraD, and TraC were again co-purified with ParM_C−FLAG_. Retention of low amounts of TraI by ParR was confirmed. No specific interaction with TraD or TraC was detected. Relaxase accessory factor TraM binds *oriT*, TraD, and the membrane (Schwab et al., 1991; Disque-Kochem and Dreiseikelmann, 1997; Lu and Frost, 2005), but antibodies to TraM did not detect co-retention of this protein with either ParM or ParR (not shown). The abundance of Par protein present in each cell extract and in the pull down fractions was quantitated using antibodies to the FLAG epitope (Figure S3). We conclude that ParR binds TraI less strongly than ParM. Moreover ParM protein in the cell binds not only TraI, but additionally the T4CP TraD and TraC. In every case, these interactions take place in the absence of an intact transfer machinery and filament formation. There is a possibility that ParM is bound by additional Tra proteins of plasmid R1 but currently we do not have antibodies available for the entire suite of purified components.

To test whether the additional binding partners, TraD and TraC, alter ParM ATPase activity, the purified proteins were assayed in combination *in vitro*. Our published (Mihajlovic et al., 2009) and unpublished (V.K.H. Rajendra and E.L.Zechner) observations show that the soluble form of TraD (lacking the N-terminal transmembrane domain; TraDΔN130) and full length TraC interact with several protein and DNA ligands *in vitro*, yet neither Tra protein increased ATP hydrolysis compared to reactions containing ParM alone (not shown).

#### Lack of ParM and ParR lowers protein translocation by the T4SS

**Figure 7:**
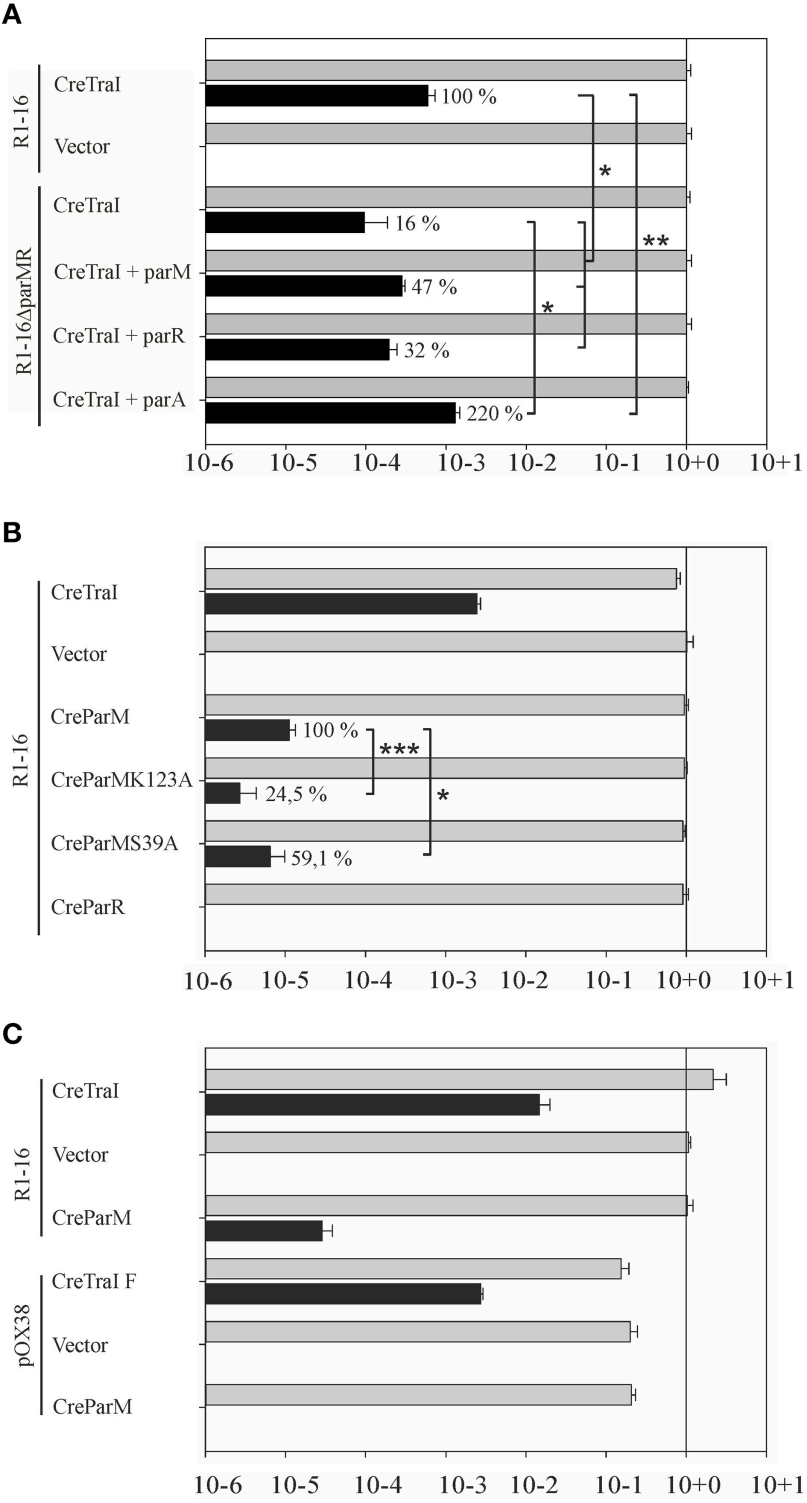
**Secretion of Cre-TraI and Cre-ParM to recipient cells. (A)** Protein translocation was detected by recombination events per donor. The frequencies of protein transfer (black bars) were normalized to conjugation efficiency for each culture (gray bars). Relative differences in Cre-TraI transfer by R1-16 *par* mutant derivatives compared to wild type (100%) are indicated. Empty complementation vector pMS119EH was used as a control. **(B)** Frequencies of translocation of the indicated Cre-fusion proteins (left) by wild type R1-16 are shown with black bars. Percent of Cre-ParM transfer observed with mutant ParM variants compared to wild type ParM (100%) is indicated. **(C)** F transfer proteins expressed by pOX38 mediate DNA transfer (gray bars) and translocation of the F TraI protein fused to Cre, but not Cre-ParM_*R*1_. Standard deviations are shown, *n* = 3, significance was determined using a onesided *t*-test, *P < 0.05; **P < 0.05; ***P < 0.005.

Some conjugative T4SS have been shown to translocate specific proteins in addition to protein-DNA adducts to recipient cells in a manner that strictly requires the T4CP. TraI is thus far the only protein known to be secreted by F-like transporters in both its nucleoprotein and DNA free forms. In both activities TraD is expected to be directly involved in recognition and binding of the (nucleo)protein substrate. We tested whether the absence of Par proteins affect protein translocation using the Cre recombinase assay for translocation (CRAfT). This technique fuses the reporter enzyme to a protein specifically secreted by the T4SS (Vergunst et al., 2000). Transfer to recipients is then quantitated by a switch in antibiotic resistance catalyzed by Cre recombination. In our assay (Lang et al., 2010) donor cells carry R1-16 to provide all the essential components for substrate recognition, conjugative DNA processing, and transport including wild type TraI protein. Here we compared the frequency of Cre-TraI transmission supported by R1-16 carrying cells to hosts carrying the double deletion R1-16Δ*parMR*. The frequency of plasmid DNA transfer was measured to provide an internal standard for the performance of the T4SS in every experiment. As shown in Figure 7A *E. coli* R1-16 transferred Cre-TraI efficiently, in good agreement with our prior results. Significantly less (six-fold) Cre-TraI was transferred in the absence of both *par* genes (R1-16Δ*parMR*). Expression of both *parM* or *parR in trans* to R1-16Δ*parMR* complemented the protein transfer defect to higher than wild type efficiency. Neither factor alone was sufficient. These data imply a role for the Par proteins in efficient TraI transfer.

### Cre-ParM fusion proteins are specifically transported to recipient cells

We next asked whether the reciprocal activity, namely the direct transfer of either Par protein by the T4SS to recipient cells, could be detected. The *cre* gene was fused 5′ to each *par* gene and translocation of ParM and ParR was analyzed by CRAfT. No Cre-ParR transfer was detected (Figure 7B). Remarkably, however, Cre-ParM translocation was measured. The observed frequency was low compared to Cre-TraI transfer. To address whether Cre-ParM transfer is the result of specific recognition, we tested ParM variants with amino acid exchanges in residues exposed on outside loops and along the surface of ParM filaments (Salje and Lowe, 2008). These residues are important to ParR binding, and crucial to stable filament formation. Both mutant variants were transferred, but CreParMK123A and CreParMS39A secretion was significantly less efficient (24.5 and 59% of wild type levels, respectively). The impact of single residue exchanges on transfer efficiency strengthens the evidence for specific ParM recognition by the T4CP. We then asked whether the highly related conjugation system of plasmid F would also mediate transfer of ParM, despite the absence of parMRC on that plasmid. For this test CRAfT assays were performed with pOX38 (Figure 7C). Although Cre fused to F TraI was efficiently secreted, we measured no Cre-ParM transfer. We conclude that translocation of ParM protein is unique for plasmid R1-16.

## Discussion

Partitioning systems are classified by their motor proteins as type I (ParA-like), type II (ParM-like), and type III (TubZ-like; Salje et al., 2010). These dynamic systems assemble into higher order structures that organize and move subcellular components. They segregate not only plasmids and bacterial chromosomes, but also partition cell organelles and proteins intracellularly (Lutkenhaus, 2012; Roberts et al., 2012; Ptacin et al., 2014; Jones and Armitage, 2015). Type I loci encode ATPases with a deviant Walker A nucleotide binding motif (Szardenings et al., 2011) The type I enzyme VirC1 of *A. tumefaciens* is required for efficient T-DNA transfer. Ground breaking work in the Christie laboratory revealed that the VirC1 motor protein promotes conjugative DNA transfer by coordinating two early steps of that process. First, VirC1 acts with auxiliary factor VirC2 to promote assembly of the relaxosome at *oriT*-like T-DNA border sequences. VirC1 then acts to spatially position the transfer intermediate at the cell pole and stimulate docking of this substrate to the T4SS channel (Atmakuri et al., 2007).

Functional links between segregation and conjugation machineries have been observed in other systems as well. The stability locus *stbABC* characterized in plasmid R388 is conserved in several Mob families (Guynet et al., 2011). Loss of R388 stability through *stbA* inactivation is caused by the plasmid's mislocalization to nucleoid-free regions of the cell. Given that the R388 T4 secretion machinery preferentially assembles at the cell poles, the accumulation of plasmid copies at the poles in the absence of StbA favors higher than normal frequencies of conjugative transfer. This functional organization thus coordinates vertical and lateral modes of plasmid R388 dissemination, i.e., conditions that jeopardize faithful plasmid inheritance are compensated by enhanced horizontal transfer. The logic of this elegant regulatory circuit is apparent but the mechanistic basis remains unknown. In particular the function of the ParA-like ATPase StbB in plasmid positioning and any possible direct contribution to conjugative transfer remain unresolved. Finally we note with interest that *parA* and *parB* of the gonococcal genetic island of *Neisseria gonorrhoeae* are involved in DNA secretion by the T4SS (Hamilton et al., 2005) but the functions performed by these proteins also await further study (Pachulec et al., 2014).

Here we report experimental evidence suggesting that the type II partitioning locus *parMRC* of plasmid R1 has been appropriated by the conjugation machinery to facilitate early steps in the assembly and function of the T4SS. Mechanistic similarities with VirC1 include the capacity of ParM and ParR to stimulate the *oriT* cleavage reaction of TraI *in vitro* and of the relaxosome *in vivo*. Although VirC1 binds to a sequence called *overdrive* adjacent to the T-DNA right border and the *oriT*-like sequence cleaved by VirD2 relaxase, ParR does not bind *oriT* of plasmid R1 and no evidence was found for an effect of the Par proteins on TraI DNA binding properties. It follows that enzyme stimulation probably occurs via direct interactions of the proteins. Indeed TraI stimulates ParM ATPase activity in the absence of DNA. Moreover the mutual stimulation of both ParM and TraI proteins was decreased when truncated versions of TraI were assayed. Finally, binding of the partner proteins *in vivo* was confirmed with pull down assays.

VirC1 is able to act as a central organizer of the relaxosome because of its DNA binding activity and because it binds pairwise with the accessory factors VirC2, VirD1, and relaxase VirD2. These interactions were detected in the absence of the Ti plasmid, therefore VirB channel components are not involved. VirC1 also associates with the polar membrane and binds the T4CP VirD4. Together these properties enable VirC1 to actively recruit the relaxosome to the cell poles and to the colocalized assembly of T4CP and the VirB T4 secretion channel.

**Figure 8:**
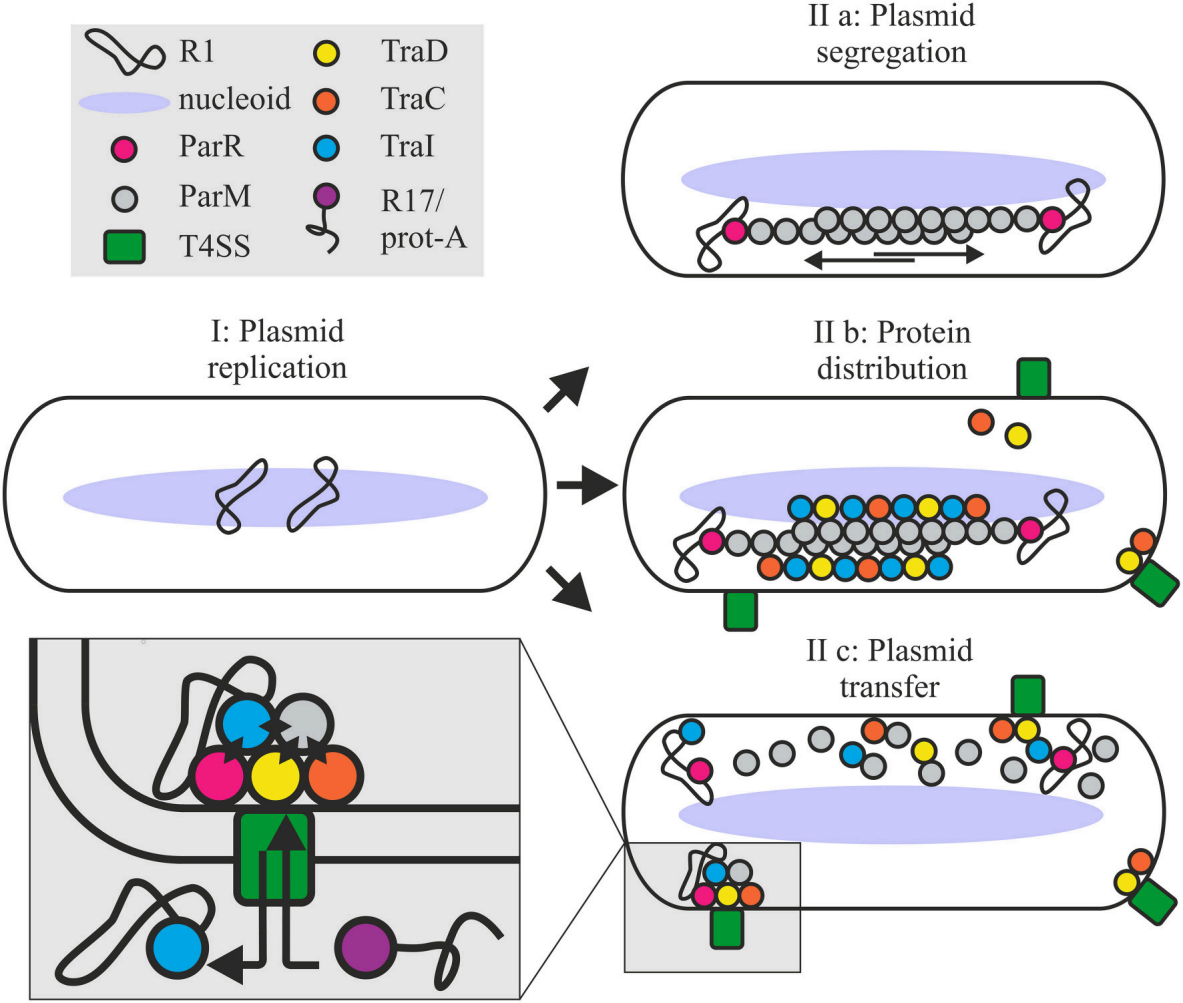
**Roles for the ParMRC system in plasmid propagation**. Newly replicated plasmids are located at midcell (I). ParR bound to centromere *parC* captures and protects the end of a growing ParM filament. Two antiparallel ParM filaments create biopolar spindles which elongate and actively segregate plasmids to opposite ends of the dividing cell (IIa). Affinity of Tra proteins for ParM concentrates these along the filament longitudinal axis promoting assembly of the T4SS (IIb). Once spindles deliver the plasmid to the cell poles ParM filaments depolymerize releasing the DNA and protein cargo. ParM and ParR proteins become integrated at the interface of relaxosome, T4CP and channel ATPase TraC (IIc) via multiple protein-protein interactions as shown by black diamonds in the expanded view. Mixed assembly of Tra proteins, Par proteins and relaxosome bring the T4SS components and or the extracellular pilus in a conformation ideally primed for conjugative DNA transfer. This optimized conformational state supports robust biofilm formation by the bacterium and renders the T4SS competent to take up the protein A-R17 RNA complex during phage infection.

In the R1 system, active relaxosomes form *in vivo* in the complete absence of *parMRC* (Karl et al., 2001). Nonetheless here we see that the absence of Par proteins decreases *in vivo* cleavage activity in the absence of conjugation and delays DNA transfer during conjugation. In the simplest model, recruitment of the R1 relaxosome to the conjugative pore would simply involve recognition of relaxase translocation signals by the T4CP receptor and docking interactions between the coupling protein and factors of the relaxosome (TraM, TraI, DNA). Alternatively however, recruitment may be enhanced by spatial determinants provided by the parMRC segregation system (Figure 8). We show that partitioning proteins ParM and ParR interact physically and functionally with several proteins of the T4 secretion machinery. Transfer proteins may have acquired an affinity for ParM to exploit the protein's cytomotive force for intracellular localization. Plasmid R1 is expected to produce very few intracellular ParM filaments that are situated close to the edge of the nucleoid (Salje et al., 2009; Bharat et al., 2015). These authors propose that the capture of plasmid DNA may take place within the nucleoid periphery. ParM filaments form continuously but are subject to dynamic instability (Garner et al., 2004, 2007). Filaments that fail to capture a ParR bound plasmid centromere rapidly disassemble. By contrast the growth of plasmid-bound filaments is stabilized long enough to push plasmids to the cell's polar extremes. Affinity of the conjugation proteins and the relaxosome for ParM should thus concentrate these along the filaments aligned on the longitudinal axis of the cell (Moller-Jensen et al., 2003; Campbell and Mullins, 2007). Once plasmids are positioned at the poles, ParM depolymerizes, allowing the plasmids to drift randomly until recaptured by a ParM filament (Campbell and Mullins, 2007). The dynamic nature of this “positioning network” should facilitate not only rapid nucleation of transporter assembly but also colocalization of T4CP and relaxosome at those sites (Figure 8). The central role of ParM in multivalent binding interactions could additionally promote efficient joining of the different subassemblies including channel ATPases and other transporter components, the T4CP and finally, docking of the relaxosome.

Importantly, our data additionally show that not only early stages of protein colocalization and *oriT*-DNA processing are enhanced, but also that the continued association of Par proteins at the interface of relaxosome, T4CP and the conjugative ATPases is important to optimal T4SS function (Figure 8). This functional interdependence governs several T4SS-mediated activities: (i) R17 phage entry via a pathway otherwise dependent on pilus conformation, and productively docked, enzymatically active complex of T4CP and relaxosome, (ii) host biofilm formation; a process relying on pilus mediated contracts with surfaces and other cells, and (iii) rapid completion of plasmid DNA transfer. Moreover, ParM and ParR interact with TraI and may directly enhance TraI protein secretion. Finally, ParM positioning at the conjugative pore and specific binding by the T4CP receptor is demonstrated by secretion of this protein to recipient cells. We have not demonstrated a specific function for ParM protein in recipient cells, but it is tempting to speculate that co-transfer of ParM may support TraI-catalyzed recircularization of T-DNA helping to stabilize this strand in the new host.

We propose a working model where positioning of the Par proteins at the T4SS channel opening induces a conformational change in the envelope-spanning complex and/or the conjugative pilus, which is important to productive biofilm formation and essential for bacteriophage R17 to penetrate the host. It follows that the altered structure generated by integration of the Par proteins seems best able to perceive or process signals conveyed by cell to surface and cell-cell contacts during biofilm formation and pilus mediated uptake of phage RNA. In the absence of this conformational shift, plasmid transfer does occur, but is indeed delayed. In summary we conclude that the simple, self-organizing ParMRC system actively promotes not only faithful vertical transmission of the low copy number plasmid R1, but also streamlines lateral spread via conjugation.

## Materials and methods

Strains: All strains used are listed in Table 1. Cultures were grown in LB-media. Antibiotic concentrations were as follows: kanamycin (Km) 50 μg/ml, chloramphenicol (Cm) 10 μg/ml, ampicillin (Amp) 100 μg/ml, tetracycline (Tc) 10 μg/ml, and streptomycin (Sm) 25 μg/ml.

**Table 1:** **Strains used in this study**.

**Strain**	**Description^a^**	**References**
*E. coli* DH5α	F−Φ80lacZΔM15 Δ(lacZYA-argF) U169 *recA1 endA1 hsdR17* (rK−, mK+) *phoA supE44* λ−*thi-1 gyrA96 relA1*	Woodcock et al., 1989
*E. coli* C41(DE3)	F-*ompT hsdS*(rB-mB-) dcm+ Tetr gal l (DE3) *endA* Hte *[*argU ileY leuW Cm^R^]^*^]	Miroux and Walker, 1996
*E. coli* BL21(DE3)	E. *coli* B F^−^ dcm *ompT hsdS*(rB− mB−) gal λ(DE3)	Studier and Moffatt, 1986
*E. coli* MS411	*ilvG rfb-50 thi*	M. Schembri, DTU, Denmark
*E. coli* CSH26Cm::LTL	Tc^R^, CSH26 *galK::cat::loxP-Tet-loxP*	Lang et al., 2010
*E. coli* DY330	W3110 *ΔlacU169 gal 490 ts λcl857 Δ (cro-bioA)*	Yu et al., 2000
*E. coli* MS614	Sm^R^, *ilvG, rfb-50, thi, rpsL*	Beutin and Achtman, 1979
*E. coli* M1174	sfrA+, P−, B1−, trp−, lys−, gal−, malA−, strR, Su−, *Δ(lac)X74*	Silverman et al., 1991

a resistances; Tc^R^, tetracycline; Sm^R^, streptomycin; Cm^R^, chloramphenicol.

Plasmids are listed in Table 2.

**Table 2:** **Plasmids used in this study**.

**Plasmids**	**Description^a^**	**Reference**
**R1 DERIVATIVES**		
R1-16miniTn5 E5	Km^R^, Cm^R^, R1-16 with miniTn5Cm inserted in the *parA* locus	Nuk et al., 2011
R1-16miniTn5 B4	Km^R^, Cm^R^, R1-16 with miniTn5Cm inserted in the *yjcA* locus	Nuk et al., 2011
R1-16Δ*parM*	Km^R^, R1-16 with gene specific *parM* null mutation	This study
R1-16Δ*parR*	Km^R^, R1-16 with gene specific *parR* null mutation	This study
R1-16Δ*parMR*	Km^R^, R1-16 with *parMR* double gene null mutation	This study
R1-16	Km^R^, IncFII, *fin-*	Goebel et al., 1977
R1-16Δ*traI*	km^r^ Tc^r^ *traI*::*tetRA*	Lang et al., 2010
pOX38	Km^R^, derivative of F	Chandler and Galas, 1983
**COMPLEMENTATION/CLONING**		
pMS_*parMR*	Amp^R^, *Eco*RI/*Bam*HI fragment of pKG321 (Gerdes et al., 1985) in pMS119HE	This study
pMS_*parM*	Amp^R^, pMS119HE with wild type R1 *parM* encoding residues 1–320	This study
pMS_*parR*	Amp^R^, pMS119HE with wild type *parR* encoding residues 1–117	This study
pMS_*parMS39A*	Amp^R^, pMS_*parM* with a S39A sense mutation	This study
pMS_*parMK123A*	Amp^R^, pMS_*parM* with a K123A sense mutation	This study
pMS119HE	Amp^R^, P_tac_ expression vector	Strack et al., 1992
pGZ119EH	Cm^R^, P_tac_ expression vector	Lessl et al., 1992
**PROTEIN TRANSLOCATION**		
CFP B	Amp^R^, Cre-fusion vector	Parker and Meyer, 2007
CFP B Sm	Sm^R^, Cre-fusion vector	Lang et al., 2010
CreParM Sm	Sm^R^, CFP B Sm with R1 *parM* encoding residues 2–320	This study
CreParMS39A Sm	Sm^R^, CreParM Sm with a S39A mutation	This study
CreParMK123A Sm	Sm^R^, CreParM Sm with a K123A mutation	This study
CreParR Sm	Sm^R^, CFP B Sm with R1 *parR* encoding residues 2–117	This study
CreTraI(3-1756) Sm	Sm^R^, CFP B Sm with R1 *traI* encoding residues 3–1756	Lang et al., 2010
CreTraIF	AmpR, *Kpn*I-*Sal*I fragment from p99I+ (Haft et al., 2006) cloned into CFP B	Lang et al., 2010
**PROTEIN EXPRESSION**		
pJSC21	Amp^R^, P_tac_ ParR expression construction	Salje and Lowe, 2008
pET3A-ParM	Amp^R^, P_tac_ ParM expression construction	This study
pHP2	Cm^R^, P_tac_ TraI expression construct, pGZ119HE with 6,1 kb *Asn*I-fragment from R1-16 in SmaI-site	Zechner et al., 1997
pGZTraD	Cm^R^, P_tac_ TraD expression vector	This study
pGZTraC	Cm^R^, P_tac_ TraC expression vector	This study
pMS-CFLAG*parM*	Amp^R^, P_tac_, C-terminal fusion of FLAG tag with *parM*	This study
pMS-NFLAG*parR*	Amp^R^, P_tac_, N-terminal fusion of FLAG tag with *parR*	This study
pMS-CFLAG*parR*	Amp^R^, P_tac_, C-terminal fusion of FLAG tag with *parR*	This study
pASKIBA7PLUSTraC	Amp^R^, P^tet^, N-terminal fusion of Strep tag with *traC*	This study
**RELAXASE/ATPASE ASSAY**		
pDE100	Amp^R^, *Bgl*II/*Pst*I fragment of R1 *oriT* in *Eco*RI/*Pst*I pBluescript II KS-	Csitkovits et al., 2004
pDE110	Amp^R^ 388-bp insert in pBluescript II KS, including a 363-bp *Cla*I/*Pst*I fragment of the R1 *traD* gene (positions 1766–2129, GenBank™ accession number AY684127)	Csitkovits et al., 2004
pBlue*parC*	Amp^R^, *EcoR*I-*parC*-*EcoR*I PCR fragment cloned via *EcoR*I restriction site into pBluscript II KS(-)	This study

a resistances; Amp^R^, ampicillin; Km^R^, kanamycin; Sm^R^, streptomycin; Cm^R^, chloramphenicol.

### DNA preparation and modification

All enzymes were used according to Manufacturer's recommendations.

Oligonucleotides are shown in Table 3.

**Table 3:** **Oligos used in this study**.

**Name**	**5′-3′ Sequence**
**CLONING**	
parMRCFW	GAC*GAATTC*CACTTTTGTTACCCGCC
parCrev	GAC*GAATTC*TTAATTTATAAAACTCCTTATGG
parM NdeI FW	TTTT*CATATG*TTGGTATTCATTGATGACG
parM BamHI REV	TTTT*GGATCC*TTAATTACCTATGAGATACATACC
ParM_SFW	ATAGTAGGTACCTTGGTATTCATTGATGACG
ParM_SRev	GCAATCGTCGACTTAATTACCTATGAGATACATACCGT
ParR_FW	ATAGTAGGTACC-ATGGACAAGCGCAGAACC
ParR_Rev	GCAATCGTCGACTTATTAATTTATTAGCTTCATCGC
parR_NheIFW	GTA*GCTAGC*ATGATGGACAAGCGCAGAACC
parR_NheIRev	GTA*GCTAGC*ATTTATTAGCTTCATCGC
parMBamHIfw	TTTT*GGATCC*ATGTTGGTATTCATTGATGACG
parMEcoRIrev	TTTT*GAATTC*TTAATTACCTATGAGATACATACC
parRBamHIfw	TTT*GGATCC*ATGATGGACAAGCGCAGAAC
parREcoRIrev	TTT*GAATTC*TTAATTTATTAGCTTCATCG
ParMloxFW	CCCAAAACATACCCAAACACACACCAAAAAAACACCATAAGGAGTTTTATAatataacttcgtataG
ParMloxRev	GTTTGATTTACATCTGGATTTAGTTTGAAGGCAATGGTTCTGCGCTTGTCCATCAGGataacttcgtataA
FW_parR_TetRA	AACCAATAACTCTCAATATGATTTAGTTAACGGTATGTATCTCATAGGTAATTACAAGAATTGCCGGCGGAT
Rev_parR_TetRA	GTTCCCTTTATCCAGCCTGATAGTGGATAAAGGGAACTCAATAATAATTGAAGGTATTTCACACCGCATAGC
SS01	GCC*GAATTC*ATGAGTTTTAACGCAAAG
SS02	CGT*GAAGCT*TTCAGAAATCATCTCCCG
parM_CFLAG_EcoRI_rev	TTATA*GAATTC*TTACTTGTCATCGTCATCCTTGTAATCATTACCTATGAGATACATACC
parR_NFLAG_BamHI_fw	TATA*GGATCC*ATGGATTACAAGGATGACGATGACAAGATGGACAAGCGCAGAAC
parR_CFLAG_EcoRI_rev	TTATA*GAATTC*TTACTTGTCATCGTCATCCTTGTAATCATTTATTAGCTTCATCGC
StrepTraCEcorIFw	AA*GAATTC*AATAACCCACTTGAGGCCG
StrepTraCHindIIIR	TTT*AAGCTT*TCATGCAACACTCCTGTATTT
**EMSA AND FLUORESCENCE INTENSITY**	
parC^*^	AAACAAAACCCAAAAACAACCC
parCcomp	GGGTTGTTTTTGGGTTTTGTTT
oriT^*^	ACCAAAAGCACCACACCCCACGCAAAAACAAG
oriTcomp	CTTGTTTTTGCGTGGGGTGTGGTGCTTTTGGT
randomseq^*^	CGAACGAGCAGTTGTTTCAGCG
randomseqcomp	CGCTGAAACAACTGCTCGTTCG
oriT17^*^	TTTGCGTGGGGTGTGGT
2 x G144C	TTTTGCGTGGGCTGTGGTCTTTGCGTGGGCTGTGGTCTTT

* = 3′-carboxytetramethyl-rhodamine (TAMRA) labeled, Restriction sites are marked in cursive, FLAG-sequences are underscored, lox-sites are in lower-case letters.

### Construction of complementation-, Cre,- and expression-plasmids

Inserts for pMS_*parM* and derivatives were amplified with parMBamHIfw/parMEcoRIrev, for pMS_*parR* with parRBamHIfw/parREcoRIrev using pMS_*parA*, pJSC1-S39A, or pJSC1-K123A as templates. Amplicons were cut with *Bam*HI/*Eco*RI and ligated with pMS119HE. N-terminal Cre fusions were constructed by inserting amplicons from templates pMS_*parM*, pMS_*parR*, or derivatives in CFB B Sm plasmid via *Kpn*I/*Sal*I. Primers for the *parM* inserts: ParM_SFW/ParM_SRev; for *parR* insert: ParR_FW/ParR_Rev. To generate the C-terminal CreParR fusion plasmid *parR* was amplified with parR_NheIFW/parR_NheIRev from template pMS_*parA*, and ligated to *Nhe*I cut CFP B. The *parM* PCR fragment from parMNdeI_fw/parMBamHI_rev and R1-16 template were cut with *Nde*I and *BamHI* and ligated to yield pet3A-*parM*. pASKIBA7PLUSTraC was generated by amplification of *traC* from R1-16 with primers StrepTraCEcorIFw and StrepTraCHindIIIR, cutting with *EcoR*I and *Hind*III and ligation in pASKIBA7PLUS.

pGZ*traD* was created by amplification of *traD* from R1-16 with primers SS01 and SS02, *EcoR*I and *Hind*III treated and inserted in pGZ119EH. To generate pGZ*traC, traC* was cut from R1-16 with *EcoR*1 and *Sma*I and ligated into pGZ119EH. pMS-CFLAGparM was constructed by amplification of *parM* with parM_CFLAG_EcoRI_rev/parMBamHI_fw, cutting with *EcoR*I and *BamH*I and ligation into pMS119EH. pMS-NFLAGparR and pMS-CLFAGparR were constructed by amplification of *parR* with primers parR_NFLAG_BamHI and parREcoRIrev or parRBamHIfw and parR_CFLAG_EcoRI_rev, respectively, restriction with *BamH*I/*EcoR*I and ligation with pMS119EH. pBlue*parC* was constructed by amplification of *parC* from R1-16 with primers parMRCFW and parCrev, restriction with *EcoR*I and ligation with pBluescript II KS(−).

### Construction of *parM, parR*, and *parMR* null derivatives

To generate R1-16Δ*parM*, primers ParMloxFW/ParMloxRev were used to amplify a *loxP*-TetRA-*loxP* cassette from *E. coli* CSH26Cm::LTL. For R1-16Δ*parR*, FW_parR_TetRA/Rev_parR_TetRA were used to amplify a tetracycline resistance cassette from pAR183 (Reisner et al., 2006). For R1-16Δ*parM R*, FW_R1parM/Rev_R1parR were used to amplify a streptomycin resistance cassette from pAH144 (Haldimann and Wanner, 2001). The amplified fragments were introduced into *E. coli* DY330 [R1-16] and integrated via homologous recombination (Reisner et al., 2002). Introduction of the CFP B plasmid into strains carrying R1-16 mutants catalyzed a Cre/*loxP* mediated recombination reaction excising the *tetRA* cassette.

### Fluorescence microscopy

Alexa488 labeled R17 phage was prepared as described (Lang et al., 2014). *E. coli* MS411 carrying R1-derivatives with and without complementation plasmids were grown to an A_600_ of 0.6–0.8, diluted in PBS to an A_600_ of 0.5 and incubated with 0.01 vol. R17 phage for 10 min at RT. Five microliters were mounted on a glass slide, pictures were taken with an Eclipse Ti fluorescence microscope (Nikon).

### Copy number determination

For quantification of apparent copy number, plasmid yields of R1 derivatives (R1-16, R1-16::B4, R1-16miniTn5 E5, or R1-16miniTn5 B4) were determined and compared to the yields of an independent replicon (pGZ119EH) as described in Nuk et al. (2011). *E. coli* M1174 carrying the desired plasmid combinations were grown in 5 ml LB medium with antibiotics. One millimolar IPTG induced *traI* (pHP2) expression.

### Mating experiments and cre recombinase assay for translocation (CRAfT)

*E. coli* MS411 carrying the plasmids were grown overnight in LB media with antibiotics at 37°C. Hundred microliters of donor cells were centrifuged for 3 min at 3000 × g, resuspended in 1 ml 0.9% NaCl, subcultured in drug free LB for 1 h at 37°C and adjusted to A_600_ 0.02. A 10-fold excess of recipient MS614 was added and the mixture was incubated for 3, 5, 15, or 30 min at 37°C without shaking. DNA transfer was stopped by vortexing for 1 min and rapid cooling on ice. Donors were selected on LB-plates with antibiotics (see Table 2) and transconjugants were selected on kanamycin (40 μg/ml) and streptomycin (25 μg/ml). Conjugation frequencies were calculated as transconjugants per donor.

CRAfT was performed as described previously (Lang et al., 2010). *E. coli* MS411 carrying the plasmids of interest and recipient CSH26Cm::LTL were used. Donors were selected on plates with antibiotics (Table 2). Transconjugants and recombinants were identified by plating serial dilutions on LB-Kan/Tc or chloramphenicol, respectively. Conjugation and protein translocation frequencies are calculated as transconjugants or recombinants per donor, respectively.

### Protein purification

TraI, TraIN309, TraIN992, TraDΔN130 were purified as described (Csitkovits et al., 2004; Mihajlovic et al., 2009; Sut et al., 2009; Lang et al., 2011).

ParR purification: 2 l *E. coli* C41(DE3) [pJSC21] were grown in LB with 100 μg/ml ampicillin (LB-Amp) at 37°C with shaking to an OD600 of 0.6. One millimolar IPTG was used for induction. After 6 h, cells were harvested (10 min, 4000 × g, 4°C), resuspended in 20 ml buffer I (50 mM Tris-HCl pH 7, 100 mM KCl, 1 mM EDTA, 1 mM DTT, 0.001% PMSF) with proteinase inhibitor cocktail (cOmplete EDTA-free, Roche) and frozen at −80°C. Cells were lysed, incubated with DNAse I (AppliChem) for 20 min on ice and centrifuged (140,000 × g, 1 h, 4°C). The cytoplasmic fraction was filtered through a 0.45 μm PVDF syringe filter and loaded (1 ml/min) on HiTrap Heparin columns equilibrated with buffer I. After washing (2 column volumes buffer I), bound protein was eluted with buffer I and a linear gradient of 0–1 M NaCl. Partially pure ParR eluted at ~300–450 mM NaCl. These fractions were pooled and dialysed overnight against 100 × vol. buffer I. Dialyzed fractions were loaded (1 ml/min) on 2 serially connected 5 ml HiTrap SP HP columns equilibrated with buffer I. After washing (2 ml/min) with 10 ml of buffer I, protein was eluted with a linear gradient of buffer I + 0–1 M NaCl. ParR eluted at ~450–500 mM NaCl. Purity was confirmed by SDS-PAGE and fractions were pooled and dialyzed overnight [100 vol. buffer II (20 mM Tris-HCl pH 9, 50 mM KCl, 1 mM EDTA, 1 mM NaN_3_)]. ParR was concentrated, adjusted to 20% glycerol and frozen at −80°C.

#### ParM purification

*E. coli* BL21 (DE3) carrying pet3A-*parM* were grown in 1l LB-Amp at 37°C. At OD_600_ 0.6 expression was induced with 1 mM IPTG. After 3 h, cells were harvested (4000 × g, 10 min, 4°C), resuspended in 20 ml buffer I (30 mM Tris-HCl pH7.5, 25 mM KCl, 1 mM MgCl_2_, 1 mM DTT, 0.001% PMSF) with protease inhibitor (cOmplete EDTA-free, Roche) and frozen at −80°C. Lysis was performed as described above. The lysate was cleared by centrifugation (21,000 × g, 1 h, 4°C) and the supernatant was precipitated by addition of solid (NH_4_)_2_SO_4_ to 30% w/v with stirring on ice (1 h). After centrifugation (77,000 × g, 1 h, 4°C) the pellet was dissolved in 20 ml buffer I and dialyzed overnight at 4°C (100 vol buffer I). Dialyzed solution was filtered (0.45 μm) loaded on 2 × 5 ml HiTrap Heparin columns equilibrated with buffer I. Flowthrough was collected and loaded onto 2 × 5 ml HitrapQ columns equilibrated with buffer I and washed with 10 ml of buffer I. Protein was eluted with a 4 step gradient with buffer I + 1 M KCl (12, 15, 20, 30%, 5 ml buffer flow between steps, followed by 10 ml isocratic elution for each step). ParM eluted at ~200 mM KCl and purity of fractions was confirmed with SDS-PAGE. Pure fractions were pooled and dialyzed overnight at 4°C (100 vol buffer I), concentrated, adjusted to 20% glycerol and frozen at −80°C.

### TraC purification

*E. coli* BL21 C41 (DE3) carrying pASKIBA7PLUSTraC were grown in 1l LB-Amp at 37°C to an OD_600_ of 0.5. Overexpression was induced by addition of anhydrotetracyclin (AHT, 0.2 mg/l). Cells were harvested after 4 h shaking at 37°C, pellets were frozen at −80°C. Pellet was resuspended in 20 ml of buffer I (50 mM Tris-Cl pH 7.7, 150 mM NaCl, 1 mM EDTA, 1 mM DTT, 1 tablet protease inhibitor (cOmplete, Roche) and lysed. The lysate was centrifuged for 45 min at 50,000 × g at 4°C. The supernatant was filtered (0.45 μm PVDF syringe filters) and loaded onto a Hitrap strep HP column pre-equilibrated with buffer I. After washing (5–10 column volumes) with buffer I, TraC was eluted with 30 ml buffer II (buffer I containing 2.5 mM desthiobiotin) in one step. Fractions containing TraC were pooled and concentrated (Amicon centrifugal filter, Millipore) and loaded onto a Hiload 16/60 Superdex 200 column. The protein was eluted with buffer III (25 mM Tris pH 7.6, 100 mM NaCl, 1 mM DTT, 1 mM MgCl_2_, 1 mM PMSF). Pure TraC fractions were pooled, concentrated and frozen at −80°C (with 20% glycerol). Identity of TraC was confirmed by mass spectrometry and apparent molecular mass 99 kDa was determined (SDS-PAGE and Coomassie staining).

### Relaxase assay

*oriT* specific cleavage activity was determined as described in Csitkovits et al. (2004). Indicated ParM and ParR concentrations were combined with 4 nM of pDE100 or pDE110 (negative control) independently or in combination, then the cleavage reaction was started by TraI (25 nM), TraIN308 (300 nM), or TraIN992 (100 nM). Statistical significance was determined using maximum stimulatory concentrations of ParM (15 nM) and ParR (15 nM) and 500 nM BSA as a negative control.

### T-strand cleavage and unwinding

Construction of heteroduplex-substrates and the assay conditions were as described in Csitkovits et al. (2004) and Sut et al. (2009). Each heteroduplex substrate G2028 or IR (1 nM) was combined with effector proteins ParM and ParR independently or in combination in concentrations that were most stimulatory for TraI in the relaxase assay. Twenty-five nanomolar TraI was added to start the reaction. Resolution and quantitation of unwound DNA product were as described (Sut et al., 2009).

### ATPase assay

Enzyme activities were measured with the Malachite Green Assay Kit (Bioassay Systems). Briefly, different ParM concentrations (0–1 mM) with and without ParR (9 mM) and *parC* (pBlueparC, 17 nM) or DNA not containing *parC* (pDE110, 17 nM) were incubated (30 mM Tris-HCl pH 7.5, 50 mM KCl, 0.2 mM MgCl_2_, 1 mM DTT, 0.1 mg/ml bovine serum albumin, 0.1 mM ATP; Jensen and Gerdes, 1997) at 30°C in a total volume of 25 μl. After 10 min, the reaction was stopped and color development after 30 min at RT was recorded at 595 nm. Stimulation of ParM ATPase activity by TraI, TraD, or TraC: 0.5 or 1 mM ParM were titrated with TraI, TraIN308, or TraIN992 (10–100 nM), TraD (20–500 nM), or TraC (0.5–8 mM), respectively, in the reaction buffer described above.

Impact of Par-Proteins on TraI ATP-hydrolysis: 1 fmol M13mp18 single-stranded phage DNA (New England BioLabs) was preincubated with ParR (0.5 mM) and ParM (9 mM) in buffer containing ATP (25 mM Tris HCl pH 7.5, 20 mM NaCl, 3 mM MgCl_2_, 5 mM DTT, 2 mM ATP). TraI addition (0–10 nM) started the reaction. Basal TraI ATPase activity was 225,892 ± 83,485 mol/h/mol.

### Electrophoretic mobility shift (EMSA)

Oligos for fluorescence studies were reconstituted in 1 × STE (10 mM Tris pH 8.0, 50 mM NaCl, 1 mM EDTA). To create dsDNA substrates 3′-TAMRA labeled oligos were mixed in a 1:1 ratio with the unlabelled complementary strand, heated for 10 min, 96°C and re-hybridized at RT for 1 h. DNA binding by ParR was tested with ssDNA and dsDNA substrates. The consensus sequence of *parC* (Moller-Jensen et al., 2003) was created with Weblogo 3.4 (Crooks et al., 2004), a random DNA sequence was created with SMS (Stothard, 2000). Briefly, ds- or ssDNA parC^*^, oriT^*^, and randomseq^*^ (all 20 nM) were titrated with ParR in a total volume of 15 μl in 10 mM Tris HCl pH7.5, 50 mM NaCl, 0.005% NONIDET P-40, 1 mM DTT, 1 mM EDTA. The reaction was incubated for 20 min at 25°C, then 2 μl 87% glycerol were added. The products were resolved on 8% PAGE-gel without SDS at 15 V/cm for 30 min. Gels were scanned with a Typhoon 9400.

### Affinity for ssDNA

TraI affinity for 3′-TAMRA oriT DNA was measured on an ATF-105 fluorometer (Aviv Biomedical, Inc., Lakewood, NJ) as described in Dostal and Schildbach (2010). Briefly, 4 nM substrates (oriT17^*^) with and without unlabelled competitor (50 nM 2 × G144C, Dostal et al., 2011) were preincubated with protein (ParR 10 nM, ParM 10 nM, 20 nM) at RT for 10 min in binding buffer (20 mM TrisHCl pH 7.5, 100 mM NaCl, 1 mM EDTA), the reaction was started with 0–100 nM TraI with a Microlab 500 titrator (Hamilton). A constant temperature of 25°C was maintained. Excitation wavelength was 520 nm, change in fluorescence intensity was followed at 580 nm. Equilibration time between each titration step was 3 min with mixing. Datapoints were averaged over 10 s. Volume correction and data fitting was done as described (Dostal and Schildbach, 2010).

### Co-immunoprecipitation of Par- with Tra- proteins

The protocol was adapted from the TrIP assay (Cascales, Christie 2004). Hundred milliliters LB was inoculated with ONCs of *E. coli* MS411 strains carrying plasmid combinations. Thirty OD of exponentially growing (LB with antibiotics, 1 mM IPTG) cells were pelleted and resuspended in 45 ml 20 mM sodium phosphate buffer (pH = 6.8) with 1% formaldehyde and incubated for 15 min at RT. Five milliliters glycine (1.2 M in 20 mM sodium phosphate buffer pH = 6.8) were added, cells were pelleted and washed with 50 ml/30 OD of buffer A (50 mM Tris-HCl pH 6.8, 100 mM NaCl). For lysis, pellet was resuspended in 1 ml buffer B (10 mM Tris-HCl pH 8.0, 10 mM MgCl_2_, 1 mg/ml lysozyme) and transferred to a 2 ml Eppendorf tube and frozen for 2 min with liquid N2, thawed on ice, frozen and thawed again. Each sample was sonicated for 10 s on ice. One, two milliliters of buffer C was added and adjusted with Triton X-100 to a final concentration of 4% and the lysate was incubated for 15 min with rotation at RT. Two hundred and thirty microliters cOmplete EDTA-free, Roche (1 tablet in 1 ml 25 mM MgCl2) was added and the mixture was incubated for 30 min at 37°C with shaking. 6.4 ml of buffer C (150 mM Tris-Hcl pH 8, 0.5 M sucrose, 10 mM EDTA) were added and insoluble material was removed by centrifugation (14,000 × g, 15 min). At this point, 200 μl aliquots of the supernatant were saved and stored at −80°C (whole cell lysate fraction, WCL). Remaining supernatant was transferred to tubes with Anti-FLAG affinity gel (90 μl/sample, A2220, Sigma) and incubated over night at 4°C. Beads were pelleted at 5000 × g, supernatant was removed. Beads were washed once with 950 μl buffer C supplemented with 1% Triton X-100 and twice with 950 μl buffer C supplemented with 0.1% Triton X-100. Immunoprecipitates (IP) were eluted by incubation of the beads for 30 min with FLAG-peptide (F3290, Sigma) at RT [80 μl FLAG-peptide (1 mg/ml in buffer C)/40 μl beads]. Beads were pelleted and the supernatant was collected (IP fraction).

### Western analysis

A_600_ 0.015–0.03 equivalents of the WCL and IP fractions were mixed with sample buffer containing DTT and SDS and resolved on SDS-PAGE (10%). Gels were blotted for 1.5 h on PVDF membranes. Blocking was done for 2 h (3% milk in TST). Detection of TraI and TraD -proteins was done with rabbit-antisera and α-rabbit HRP-conjugated antibody (7074S, Cellsignalling). Affinity purified TraC antibodies raised against TraC were produced by immunoGlobe. FLAG-tagged proteins were detected by HRP-conjugated α-FLAG antibody (A8592, Sigma). After washing (3 × 5 min) with 1 × TST, secondary antibody was incubated for 1 h. Blot development was done with ECL (Clarity Western, Bio-Rad).

### Computer programs

ImageJ (Schneider et al., 2012) was used to quantify bands in gel-electrophoresis assays and SigmaPlot 12.2.0.45 and Qtiplot 0.9.8.3.3 were used to plot data.

## Author contributions

CG, SL, JS, and EZ designed the research. CG, SL, VR, SR, and MN did the experiments. CG, SL, JS, and EZ analyzed data. CG and EZ wrote the paper.

### Conflict of interest statement

The authors declare that the research was conducted in the absence of any commercial or financial relationships that could be construed as a potential conflict of interest.
