# Supplementary material for: Conjugative DNA Transfer Is Enhanced by Plasmid R1 Partitioning Proteins
Source: Front Mol Biosci. 2016 Jul 19;3:32. doi: 10.3389/fmolb.2016.00032 (PMC4949242; doi:10.3389/fmolb.2016.00032)
Supplement: Supplementary file 1 [file DataSheet1.PDF]

Supplementary information:

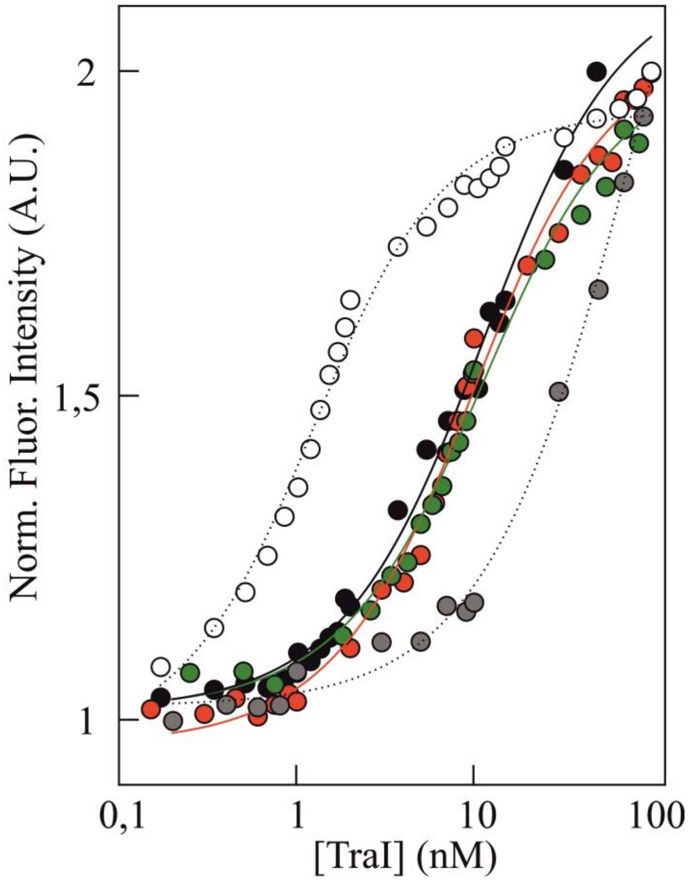

Figure S1: Par-Proteins do not change TraI affinity for DNA. K_Ds for TraI (full black circle), TraIN308 (empty circle) and TraIΔN308 (grey circle) were determined. The presence of ParM or ParR (both 10nM) did not change affinity of TraI(+ParM: green circle, +ParR: red circle) for DNA.

Because ParR itself did not specifically bind to *oriT*, we next asked whether the Par proteins alter TraI-DNA interactions. We tested two hypotheses: Par-mediated stimulation of relaxase activity is due to (i) a higher rate of TraI association with the substrate or (ii) a stabilization of the product in a cleaved state. Fluorescence intensity measurements of TraI association with a 3'-TAMRA labelled 17mer *nic*-substrate have been described in detail (Stern and Schildbach, 2001; Harley et al., 2002; Williams and Schildbach, 2006; Hekman et al., 2008; Dostal and Schildbach, 2010). We determined a stable K_d for the same 17mer *nic*-substrate of TraI (4,05 +/- 0,32), for the purified relaxase (TraIN308, K_D = 0,87+/-0,19) and the helicase (TraIΔN308, K_D = 82,56+/-13,06). We attribute the different K_D's of TraI and TraIN308 to an interference of the helicase- with the relaxase associated binding site of the full length protein, which is absent in the purified relaxase domain. We predicted that a gain in affinity of the relaxase for DNA due to TraI/Par-protein interaction would be reflected in a

shift from the characteristic K_D of TraI for the *nic*-substrate to values resembling the K_D of TraIN308. To explore the second hypothesis we asked whether the Par proteins could alter the occupation of TraI's distinct ssDNA binding sites. One binding site is associated specifically with the relaxase and the second with the helicase domain. Occupation of these sites is mutually exclusive and current models predict that the DNA is handed progressively from the first to the latter to initiate DNA unwinding and transfer (Sut et al., 2009; Dostal and Schildbach, 2010). Stabilisation of the nicked conformation could thus be achieved by shifting DNA from the relaxase-associated site to the helicase-associated binding site.

To test whether the Par proteins alter TraI-DNA binding we measured TraI association with the 17mer *nic*-substrate (oriT17*) in the presence of an excess of unlabelled competitor oligo (2xG144C) preferentially bound by the helicase-associated site (Dostal and Schildbach, 2010). No significant variations in signal intensity were induced by the additional presence of 10nM of ParR (K_D = 7,46 +/- 0,87) or ParM (K_D = 8,26 +/- 1,25). We conclude that the partitioning factors do not stimulate the relaxase reaction by altering the enzyme's DNA binding properties.

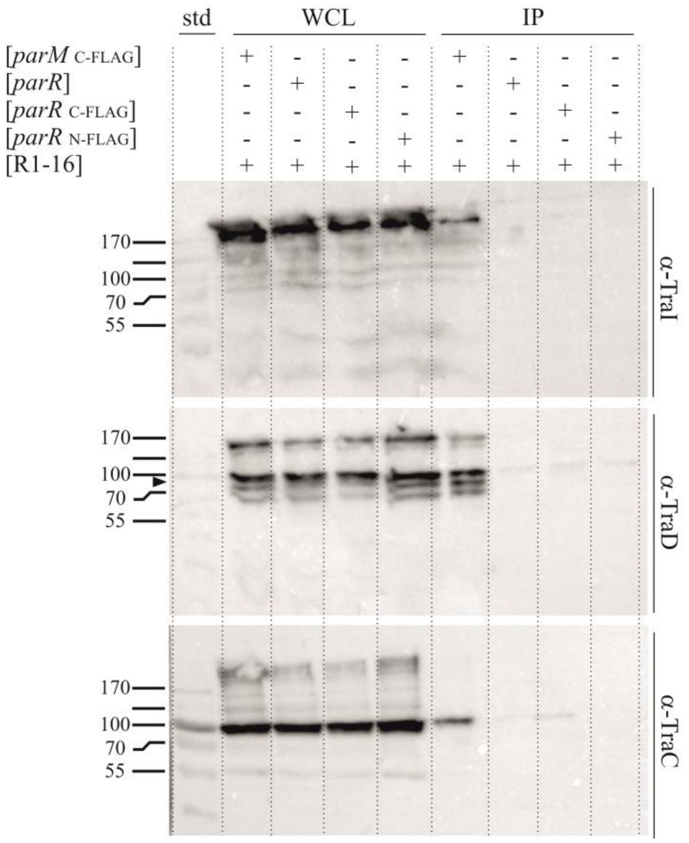

Figure S2: Protein-Protein interactions among the Par and Tra proteins. Proteins in whole cell lysates (WCL) or co-retained by FLAG-pull down (IP) were analyzed by western blotting and immunostaining. *E. coli* MS411 cells carry R1-16 for normal expression levels of Tra proteins and a second expression vector for the indicated *par* alleles. Tra proteins detected by pull down of the FLAG-tagged Par proteins are shown to the right. Band corresponding to TraD (83kDa) is indicated. Molecular mass markers (std) with sizes (kDa) are shown (left).

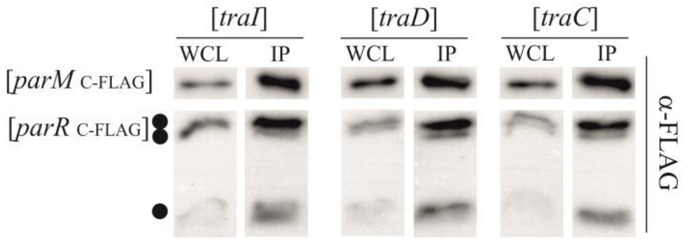

Figure S3: Detection of FLAG-Par fusion protein. *E. coli* MS411 cells carrying plasmids with indicated tra-genes and coexpressing FLAG-tagged par- alleles were treated with formaldehyde to cross-link interacting proteins. To verify the presence of FLAG-tagged Par proteins, antibodies to the FLAG epitope were used to detect protein in whole cell lysates (WCL) and the fractions retained on FLAG affinity beads (IP). Black dots indicate ParR mono- or dimers (13kDA or 26kDa, respectively).

Supplementary References:

Dostal, L., and Schildbach, J. F. (2010). Single-stranded DNA binding by F TraI relaxase and helicase domains is coordinately regulated. *J. Bacteriol.* 192, 3620–3628. doi: 10.1128/JB.00154-10

Harley, M. J., Toptygin, D., Troxler, T., and Schildbach, J. F. (2002). R150A mutant of F TraI relaxase domain: reduced affinity and specificity for single-stranded DNA and altered fluorescence anisotropy of a bound labeled oligonucleotide. *Biochemistry* 41, 6460–6468.

Hekman, K., Guja, K., Larkin, C., and Schildbach, J. F. (2008). An intrastrand three-DNA-base interaction is a key specificity determinant of F transfer initiation and of F TraI relaxase DNA recognition and cleavage. *Nucleic Acids Res.* 36, 4565–4572. doi: 10.1093/nar/gkn422

Stern, J. C., and Schildbach, J. F. (2001). DNA recognition by F factor TraI36: highly sequence-specific binding of single-stranded DNA. *Biochemistry* 40, 11586–11595.

Sut, M. V., Mihajlovic, S., Lang, S., Gruber, C. J., and Zechner, E. L. (2009). Protein and DNA effectors control the TraI conjugative helicase of plasmid R1. *J. Bacteriol.* 191, 6888–6899. doi: 10.1128/JB.00920-09

Williams, S. L., and Schildbach, J. F. (2006). Examination of an inverted repeat within the F factor origin of transfer: context dependence of F TraI relaxase DNA specificity. *Nucleic Acids Res.* 34, 426–435. doi: 10.1093/nar/gkj444
